# Draft Genome Sequence of the Oleaginous Yeast *Cryptococcus albidus* var. *albidus*

**DOI:** 10.1128/genomeA.00390-16

**Published:** 2016-05-19

**Authors:** Shashwat Vajpeyi, Kartik Chandran

**Affiliations:** Department of Earth and Environmental Engineering, Columbia University, New York, New York, USA

## Abstract

We report the complete draft genome sequence of *Cryptococcus albidus* var. *albidus*, an oleaginous yeast, which can utilize various organic carbon sources for lipid synthesis. Availability of this genome will help elucidate factors driving lipid accumulation in *C. albidus* and contribute toward bioprocess development and optimization for engineered lipid production.

## GENOME ANNOUNCEMENT

Here, we present the draft genome sequence of *Cryptococcus albidus* var. *albidus*, an oleaginous yeast that can utilize a wide variety of organic carbon sources such as glucose (1), acetic acid (2), and butyric acid (3) for the synthesis of lipids, which can be used for the commercial production of biodiesel and commodity chemicals. Notwithstanding documented studies on factors promoting lipid production and accumulation in *C. albidus*, a lack of knowledge relating to the associated metabolic pathways and mechanisms has limited further efforts toward bioprocess engineering and system optimization. We expect that the presented genome sequence will significantly enhance our understanding of lipid production and accumulation in *C. albidus* and contribute to efforts for engineered production of related biofuels and bio-based chemicals.

*Cryptococcus albidus* (ATCC 10672) was obtained from the American Type Culture Collection (ATCC, Manassas, VA, USA) and cultivated on YM (yeast and mold) medium in a 100-mL shake flask culture at room temperature (T = 25°C). Cells were harvested from 100 µL culture broth (~2 × 10^7^ cells) by centrifuging at 16,000 × *g* for 10 min at 4°C, followed by automated extraction of genomic DNA from the resulting pellet using Qiacube.

A 400-bp genome library of *C. albidus* was prepared and sequenced using Ion-torrentPGM. The raw sequence data contained 3,163,623 reads with median length of 362 bp, resulting in 44.8-fold coverage. The raw sequence reads were assembled using SPADES assembler version 3.1.0 (4) with a *k*-mer size of 21. This led to a genome assembly containing 834 contigs (sequence length >500 bp). The total consensus genome length was 24,807,186 bp with a median contig length (*N*_50_) of 114,383 bp and a G+C% content of 52.7%.

Open reading frames (ORFs) were assigned using the evidence-based annotation pipeline AUGUSTUS (5), trained using *Cryptococcus neoformans gattii*, the closest organism for which the fully sequenced genome is available. A total of 8,637 putative ORFs were identified, and 6,110 known proteins were annotated using NCBI BLASTp (6) (protein vs. protein alignment). Additional functional annotation performed using BlastKOALA (7) revealed essential pathways for metabolism of various carbon sources (including glucose, sucrose, citric, acetic, propionic and butyric acids) and for accumulation of carbon compounds (including lipids and glycogen). Inferred pathways also included uptake and assimilation of various nitrogen sources (ammonia, nitrate, nitrite, and urea) and the capability of assimilative reduction of sulfate to sulfide. Specifically pertaining to oleaginity in *C. albidus*, we identified two copies of the *acly* gene and 19 copies of the *me2* and *mdh2* genes coding for enzymes ATP citrate lyase and malate dehydrogenase, respectively, which ensure the continuous cytosolic availability of acetyl-CoA and NADPH for lipid biosynthesis (8). We also identified seven copies of genes *idh1, idh2*, and *idh3* coding for NAD^+^- and NADP^+^-dependent isocitrate dehydrogenase, which diverts carbon flow away from the citric acid cycle to lipid biosynthesis under nitrogen limitation (9), and eight copies of *acc1*, coding for acetyl-coA carboxylase, which catalyzes carboxylation of acetyl-coA to malonyl-coA, the rate-limiting step in lipid biosynthesis*. C. albidus* contains both *fas2* and *fas1* genes coding for α- and β-subunits of cytosolic type I fatty-acid synthase, which is typical in yeasts (10).

### Nucleotide sequence accession numbers. 

The draft genome sequence has been deposited to DDBJ/EMBL/GenBank under the accession number LKPZ00000000. The version discussed in this paper is the first version, LKPZ00000000.1.
